# Generalizations of some fractional integral inequalities via generalized Mittag-Leffler function

**DOI:** 10.1186/s13660-017-1389-9

**Published:** 2017-05-23

**Authors:** Ghulam Abbas, Khuram Ali Khan, Ghulam Farid, Atiq Ur Rehman

**Affiliations:** 1 Department of Mathematics, Government College Bhalwal, Sargodha, Pakistan; 2 Department of Mathematics, University of Sargodha, Sargodha, Pakistan; 3 Department of Mathematics, COMSATS Institute of Information Technology, Attock, Pakistan

**Keywords:** 26A51, 26A33, 33E12, convex function, Hadamard inequality, Mittag-Leffler function, Riemann-Liouville fractional integral

## Abstract

Fractional inequalities are useful in establishing the uniqueness of solution for partial differential equations of fractional order. Also they provide upper and lower bounds for solutions of fractional boundary value problems. In this paper we obtain some general integral inequalities containing generalized Mittag-Leffler function and some already known integral inequalities have been produced as special cases.

## Introduction

Inequalities play a vital role in both pure and applied mathematics. Specially, inequalities involving the derivative and the integral of functions are very captivating for researchers. Convex functions play an important role in the study of inequalities in all kinds of mathematical analysis.

### Definition 1

A function $f: I\rightarrow\mathbb{R}$, where *I* is an interval in $\mathbb{R}$, is said to be a convex function if

$$f\bigl(tx+(1-t)y\bigr)\leq tf(x)+(1-t)f(y) $$

holds for $t\in[0,1]$ and $x,y\in I$.

### Theorem 1.1

*Let a function* $f: I\rightarrow\mathbb{R}$ *be convex on* *I*. *Then we have*

$$f \biggl( \frac{a+b}{2} \biggr) \leq\frac{1}{b-a} \int_{a}^{b}f(x)\,dx \leq\frac{f(a)+f(b)}{2}, $$

*where* $a,b\in I$, $a< b$.

In the literature this inequality is known as the Hadamard inequality.

Recently, a number of researchers have taken great interest in establishing the Hadamard type inequalities for fractional integral operators of different kinds in the diverse field of fractional calculus. For example one may refer to [1–6].

## Fractional derivative and integral operators

Fractional calculus is a theory of integral and differential operators of non-integral order. Many mathematicians, like Liouville, Riemann and Weyl, made major contributions to the theory of fractional calculus. The study on the fractional calculus continued with the contributions from Fourier, Abel, Lacroix, Leibniz, Grunwald and Letnikov. For details, see [2, 3, 7]. A first formulation of an integral operator of fractional order in reliable form is named the Riemann-Liouville fractional integral operator.

### Definition 2

[2, 8]

Let $f\in L[a,b]$. Then Riemann-Liouville fractional integrals of *f* of order $\nu>0$ with $a\geq0$ are defined by

$$I_{a^{+}}^{\nu}f(x)=\frac{1}{\Gamma(\nu)} \int_{a}^{x}(x-t)^{\nu-1}f(t)\,dt,\quad x> a, $$

and

$$I_{b_{-}}^{\nu}f(x)=\frac{1}{\Gamma(\nu)} \int_{x}^{b}(t-x)^{\nu-1}f(t)\,dt,\quad x< b, $$

where

$$\Gamma(\nu)= \int_{0}^{\infty}t^{\nu-1}e^{-t}\,dt, $$

it is clear that $\Gamma(\nu+1)=\nu\Gamma(\nu)$.

### Definition 3

[9]

Let $f\in L[a,b]$. Then Riemann-Liouville *k*-fractional integrals of *f* of order $\nu>0$ with $a\geq0$ are defined by

$$I_{{a}^{+}}^{\nu,k}f(x)=\frac{1}{k\Gamma_{k}(\nu)} \int_{a}^{x}(x- \mu)^{\frac{\nu}{k}-1}f(\mu)\,d\mu,\quad x> a, $$

and

$$I_{{b}_{-}}^{\nu,k}f(x)=\frac{1}{k\Gamma_{k}(\nu)} \int_{x}^{b}(x- \mu)^{\frac{\nu}{k}-1}f(\mu)\,d\mu,\quad x< b, $$

where

$$\Gamma_{k}(\nu)= \int_{0}^{\infty}t^{\nu-1}e^{-\frac{t^{k}}{k}}\,dt. $$

Also $\Gamma(\nu)=\lim_{k\rightarrow1}\Gamma_{k}(\nu)$, $\Gamma_{k}(\nu)=k^{\frac{\nu}{k}-1}\Gamma(\frac{\nu}{k})$ and $\Gamma_{k}(\nu+k)=\nu\Gamma_{k}(\nu)$.

Actually, these forms of fractional integral operators have been formulated due to the work of Sonin [10], Letnikov [11] and then by Laurent [12]. Now a days a variety of fractional integral operators are under discussion. Many generalized fractional integral operators also take part in generalizing the theory of fractional integral operators [2, 3, 6, 8, 9, 13–15].

### Definition 4

[15]

Let *μ*, *ν*, *k*, *l*, *γ* be positive real numbers and $\omega\in\mathbb{R}$. Then the generalized fractional integral operator containing the generalized Mittag-Leffler function $\epsilon_{\mu,\nu,l,\omega,a^{+}}^{\gamma,\delta,k}$ and $\epsilon_{\mu,\nu,l,\omega,b^{-}}^{\gamma,\delta,k}$ for a real valued continuous function *f* is defined by

1 $$ \bigl( \epsilon_{\mu,\nu,l,\omega,a^{+}}^{\gamma ,\delta,k}f \bigr) (x)= \int_{a}^{x}(x-t)^{\nu-1}E_{\mu,\nu,l}^{\gamma,\delta,k} \bigl(\omega(x-t)^{\mu}\bigr)f(t)\,dt $$

and

$$\bigl( \epsilon_{\mu,\nu,l,\omega,b^{-}}^{\gamma,\delta,k}f \bigr) (x)= \int_{x}^{b}(t-x)^{\nu-1}E_{\mu,\nu,l}^{\gamma,\delta,k} \bigl(\omega(t-x)^{\mu}\bigr)f(t)\,dt, $$

where the function $E_{\mu,\nu,l}^{\gamma,\delta,k}$ is the generalized Mittag-Leffler function defined as

2 $$ E_{\mu,\nu,l}^{\gamma,\delta,k}(t)= \sum _{n=0}^{\infty}\frac{( \gamma)_{kn} t^{n}}{\Gamma(\mu n+\nu) (\delta)_{ln}}, $$

$(a)_{n}$ is the Pochhammer symbol, it is defined as $(a)_{n}= a(a+1)(a+2)\ldots(a+n-1), (a)_{0}=1$. If $k=l=1$ in (1), then the integral operator $\epsilon_{\mu,\nu,l,\omega,a^{+}}^{ \gamma,\delta,k}$ reduces to an integral operator $\epsilon_{\mu,\nu,1,\omega,a^{+}}^{\gamma,\delta,1}$ containing generalized Mittag-Leffler function $E_{\mu,\nu,1}^{\gamma,\delta,1}$ introduced by Srivastava and Tomovski in [6]. Along with $k=l=1$, in addition if $\delta=1$ then (1) reduces to an integral operator defined by Prabhaker in [4] containing Mittag-Leffler function $E_{\mu,\nu}^{\gamma}$. For $\omega=0$ in (1), the integral operator $\epsilon_{\mu,\nu,l,\omega,a^{+}} ^{\gamma,\delta,k}$ reduces to the Riemann-Liouville fractional integral operator [15].

In [6, 15] the properties of the generalized integral operator and the generalized Mittag-Leffler function are studied in brief. In [15] it is proved that $E_{\mu,\nu,l}^{\gamma, \delta,k}(t)$ is absolutely convergent for all $t\in\mathbb{R}$ where $k< l+\mu$.

Since

$$\bigl\vert E_{\mu,\nu,l}^{\gamma,\delta,k}(t)\bigr\vert \leq\sum _{n=0}^{\infty} \biggl\vert \frac{(\gamma)_{kn} t^{n}}{\Gamma(\mu n+\nu) (\delta)_{ln}}\biggr\vert , $$

with $\sum_{n=0}^{\infty}\vert\frac{(\gamma)_{kn} t^{n}}{\Gamma (\mu n+\nu) (\delta)_{ln}}\vert=S$, we have

$$\bigl\vert E_{\mu,\nu,l}^{\gamma,\delta,k}(t)\bigr\vert \leq S. $$

We use this definition of *S* in the sequel in our results.

A lot of authors presently are working on inequalities involving fractional integral operators, for example the versions of Riemann-Liouville, Caputo, Hillfer, Canvati etc. In fact fractional integral inequalities are useful in establishing the uniqueness of solutions for partial differential equations of fractional order, also they provide upper and lower bounds for solutions of fractional boundary value problems.

In this paper we give some integral inequalities for a generalized fractional integral operator containing the generalized Mittag-Leffler function which are generalizations of several results proved in [16–19].

The following result was obtained by Sarikaya *et al.* in [19].

### Theorem 2.1

*Let* $f:[a,b]\rightarrow\mathbb{R}$ *be a positive and convex function with* $0 \leq a< b$. *If* $f \in L[a,b]$, *then the following inequalities for a fractional integral hold*:

$$f \biggl( \frac{a+b}{2} \biggr) \leq\frac{2^{\nu-1}\Gamma(\nu +1)}{(b-a)^{ \nu}} \bigl[ I_{(\frac{a+b}{2})^{+}}^{\nu}f(b)+I_{(\frac{a+b}{2})^{-}} ^{\nu }f(a) \bigr] \leq\frac{f(a)+f(b)}{2}. $$

## Main results

First of all we establish the following result which would be helpful to obtain the main result.

### Lemma 3.1

*Let* $f:I\rightarrow\mathbb{R}$ *be a differentiable mapping on* *I*, $a,b\in I$ *with* $a< b$ *and let* $g:[a,b]\rightarrow\mathbb{R}$ *be continuous on* $[a,b]$. *If* $f'\in L[a,b]$, *then the following equality holds*:

3 $$\begin{aligned} & \biggl( \int_{a}^{b}g(s)E_{\mu,\nu,l}^{\gamma,\delta,k} \bigl(\omega s ^{\mu}\bigr)\,ds \biggr) ^{\nu}\bigl[f(a)+f(b) \bigr]-\nu \int_{a}^{b} \biggl( \int_{a} ^{t}g(s)E_{\mu,\nu,l}^{\gamma,\delta,k} \bigl(\omega s^{\mu}\bigr)\,ds \biggr) ^{\nu-1} \\ & \qquad {}\times g(t)E_{\mu,\nu,l}^{\gamma,\delta,k}\bigl(\omega t^{\mu}\bigr)f(t)\,dt \\ &\qquad {} - \nu \int_{a}^{b} \biggl( \int_{t}^{b}g(s)E_{\mu,\nu,l}^{\gamma, \delta,k}\bigl( \omega s^{\mu}\bigr)\,ds \biggr) ^{\nu-1}g(t)E_{\mu,\nu,l}^{ \gamma,\delta,k} \bigl(\omega t^{\mu}\bigr)f(t)\,dt \\ &\quad = \int_{a}^{b} \biggl( \int_{a}^{t}g(s)E_{\mu,\nu,l}^{\gamma,\delta,k} \bigl(\omega s^{\mu}\bigr)\,ds \biggr) ^{\nu}f'(t)\,dt \\ &\qquad {}- \int_{a}^{b} \biggl( \int_{t}^{b}g(s)E_{\mu,\nu,l}^{\gamma,\delta,k} \bigl(\omega s^{\mu}\bigr)\,ds \biggr) ^{\nu}f'(t)\,dt. \end{aligned}$$

### Proof

To prove this lemma, we have

4 $$\begin{aligned} & \int_{a}^{b} \biggl( \int_{a}^{t}g(s)E_{\mu,\nu,l}^{\gamma,\delta,k} \bigl( \omega s^{\mu}\bigr)\,ds \biggr) ^{\nu}f'(t)\,dt \\ &\quad = \biggl( \int_{a}^{b}g(s)E_{\mu,\nu,l}^{\gamma,\delta,k} \bigl(\omega s^{\mu}\bigr)\,ds \biggr) ^{\nu}f(b)-\nu \int_{a}^{b} \biggl( \int_{a}^{t}g(s)E _{\mu,\nu,l}^{\gamma,\delta,k} \bigl(\omega s^{\mu}\bigr)\,ds \biggr) ^{\nu-1} \\ &\qquad {}\times g(t)E_{\mu,\nu ,l}^{\gamma,\delta,k} \bigl(\omega t^{\mu}\bigr)f(t)\,dt. \end{aligned}$$

Similarly

5 $$\begin{aligned} & \int_{a}^{b} \biggl( \int_{t}^{b}g(s)E_{\mu,\nu,l}^{\gamma,\delta,k}\bigl( \omega s^{\mu}\bigr)\,ds \biggr) ^{\nu}f'(t)\,dt \\ &\quad =- \biggl( \int_{a}^{b}g(s)E_{\mu,\nu,l}^{\gamma,\delta,k} \bigl(\omega s^{\mu}\bigr)\,ds \biggr) ^{\nu}f(a)+\nu \int_{a}^{b} \biggl( \int_{t}^{b}g(s)E _{\mu,\nu,l}^{\gamma,\delta,k} \bigl(\omega s^{\mu}\bigr)\,ds \biggr) ^{\nu-1} \\ &\qquad {}\times g(t)E_{\mu,\nu ,l}^{\gamma,\delta,k} \bigl(\omega t^{\mu}\bigr)f(t)\,dt. \end{aligned}$$

Subtracting equation (5) from (4), we get (3). □

By using Lemma 3.1 we prove the following theorem.

### Theorem 3.2

*Let* $f:I\rightarrow\mathbb{R}$ *be a differentiable function on* *I*, $a,b\in I$ *with* $a< b$ *and also let* $g:[a,b]\rightarrow\mathbb{R}$ *be continuous function on* $[a,b]$. *If* $\vert f'\vert$ *is convex function on* $[a,b]$, *then the following inequality holds for* $k< l+\mu$:

$$\begin{aligned} &\biggl\vert \biggl( \int_{a}^{b}g(s)E_{\mu,\nu,l}^{\gamma,\delta,k} \bigl( \omega s^{\mu}\bigr)\,ds \biggr) ^{\nu}\bigl[f(a)+f(b) \bigr]-\nu \int_{a}^{b} \biggl( \int_{a}^{t}g(s)E_{\mu,\nu,l}^{\gamma,\delta,k} \bigl(\omega s^{\mu}\bigr)\,ds \biggr) ^{\nu-1} \\ &\qquad {}\times g(t)E_{\mu,\nu,l}^{\gamma,\delta,k}\bigl(\omega t^{\mu} \bigr)f(t)\,dt \\ &\qquad {} -\nu \int_{a}^{b} \biggl( \int_{t}^{b}g(s)E_{\mu,\nu,l}^{\gamma, \delta,k}\bigl( \omega s^{\mu}\bigr)\,ds \biggr) ^{\nu-1}g(t)E_{\mu,\nu,l}^{ \gamma,\delta,k} \bigl(\omega t^{\mu}\bigr)f(t)\,dt\biggr\vert \\ &\quad \leq\frac{(b-a)^{\nu+1}\Vert g\Vert _{\infty}^{\nu}S^{\nu }}{\nu+1}\bigl[\bigl\vert f'(a)\bigr\vert + \bigl\vert f'(b)\bigr\vert \bigr], \end{aligned}$$

*where* $\Vert g \Vert _{\infty}= \sup_{t\in[a,b]}\vert g(t)\vert$.

### Proof

By Lemma 3.1, we have

6 $$\begin{aligned} &\biggl\vert \biggl( \int_{a}^{b}g(s)E_{\mu,\nu,l}^{\gamma,\delta,k} \bigl( \omega s^{\mu}\bigr)\,ds \biggr) ^{\nu}\bigl[f(a)+f(b) \bigr]-\nu \int_{a}^{b} \biggl( \int_{a}^{t}g(s)E_{\mu,\nu,l}^{\gamma,\delta,k} \bigl(\omega s^{\mu}\bigr)\,ds \biggr) ^{\nu-1} \\ &\qquad {}\times g(t)E_{\mu,\nu,l}^{\gamma,\delta,k}\bigl(\omega t^{\mu} \bigr)f(t)\,dt \\ &\qquad {} -\nu \int_{a}^{b} \biggl( \int_{t}^{b}g(s)E_{\mu,\nu,l}^{\gamma, \delta,k}\bigl( \omega s^{\mu}\bigr)\,ds \biggr) ^{\nu-1}g(t)E_{\mu,\nu,l}^{ \gamma,\delta,k} \bigl(\omega t^{\mu}\bigr)f(t)\,dt\biggr\vert \\ &\quad \leq \int_{a}^{b}\biggl\vert \int_{a}^{t}g(s)E_{\mu,\nu,l}^{\gamma, \delta,k} \bigl(\omega s^{\mu}\bigr)\,ds \biggr\vert ^{\nu}\bigl\vert f'(t)\bigr\vert \,dt \\ &\qquad {}+ \int_{a}^{b} \biggl\vert \int_{t}^{b}g(s)E_{\mu,\nu,l}^{\gamma,\delta,k}\bigl( \omega s ^{\mu}\bigr)\,ds \biggr\vert ^{\nu}\bigl\vert f'(t)\bigr\vert \,dt. \end{aligned}$$

By using $\Vert g \Vert _{\infty}= \sup_{t\in[a,b]}\vert g(t)\vert$ and absolute convergence of the generalized Mittag-Leffler function, we have

7 $$\begin{aligned} &\biggl\vert \biggl( \int_{a}^{b}g(s)E_{\mu,\nu,l}^{\gamma,\delta,k} \bigl( \omega s^{\mu}\bigr)\,ds \biggr) ^{\nu}\bigl[f(a)+f(b) \bigr]-\nu \int_{a}^{b} \biggl( \int_{a}^{t}g(s)E_{\mu,\nu,l}^{\gamma,\delta,k} \bigl(\omega s^{\mu}\bigr)\,ds \biggr) ^{\nu-1} \\ & \qquad {}\times g(t)E_{\mu,\nu,l}^{\gamma,\delta,k}\bigl(\omega t^{\mu} \bigr)f(t)\,dt \\ &\qquad {} -\nu \int_{a}^{b} \biggl( \int_{t}^{b}g(s)E_{\mu,\nu,l}^{\gamma, \delta,k}\bigl( \omega s^{\mu}\bigr)\,ds \biggr) ^{\nu-1}g(t)E_{\mu,\nu,l}^{ \gamma,\delta,k} \bigl(\omega t^{\mu}\bigr)f(t)\,dt\biggr\vert \\ &\quad \leq \Vert g\Vert _{\infty}^{\nu}S^{\nu} \biggl[ \int_{a}^{b}(t-a)^{\nu}\bigl\vert f'(t)\bigr\vert \,dt+ \int_{a}^{b}(b-t)^{\nu}\bigl\vert f'(t)\bigr\vert \,dt \biggr] . \end{aligned}$$

Since $\vert f'\vert$ is convex function, it can be written as

8 $$ \bigl\vert f'(t)\bigr\vert \leq \frac{b-t}{b-a}\bigl\vert f'(a)\bigr\vert +\frac{t-a}{b-a} \bigl\vert f'(b)\bigr\vert $$

for $t\in[a,b]$.

Using (8) in (7), we have

9 $$\begin{aligned} &\biggl\vert \biggl( \int_{a}^{b}g(s)E_{\mu,\nu,l}^{\gamma,\delta,k} \bigl( \omega s^{\mu}\bigr)\,ds \biggr) ^{\nu}\bigl[f(a)+f(b) \bigr]-\nu \int_{a}^{b} \biggl( \int_{a}^{t}g(s)E_{\mu,\nu,l}^{\gamma,\delta,k} \bigl(\omega s^{\mu}\bigr)\,ds \biggr) ^{\nu-1} \\ & \qquad {}\times g(t)E_{\mu,\nu,l}^{\gamma,\delta,k}\bigl(\omega t^{\mu} \bigr)f(t)\,dt \\ &\qquad {}-\nu \int_{a}^{b} \biggl( \int_{t}^{b}g(s)E_{\mu,\nu,l}^{\gamma, \delta,k}\bigl( \omega s^{\mu}\bigr)\,ds \biggr) ^{\mu-1}g(t)E_{\mu,\nu,l}^{ \gamma,\delta,k} \bigl(\omega t^{\mu}\bigr)f(t)\,dt\biggr\vert \\ &\quad \leq \Vert g\Vert _{\infty}^{\nu}S^{\nu} \biggl[ \int_{a}^{b}(t-a)^{\nu} \biggl( \frac{b-t}{b-a}\bigl\vert f'(a)\bigr\vert +\frac{t-a}{b-a} \bigl\vert f'(b)\bigr\vert \biggr)\,dt \\ & \qquad {} + \int_{a}^{b}(b-t)^{\nu} \biggl( \frac{b-t}{b-a}\bigl\vert f'(a)\bigr\vert + \frac{t-a}{b-a}\bigl\vert f'(b)\bigr\vert \biggr)\,dt \biggr] . \end{aligned}$$

After simplification of inequality (9) we get the result. □

### Remark 3.3

By giving particular values to parameters in the generalized Mittag-Leffler function several fractional integral inequalities can be obtained for corresponding fractional integrals. For example for the Riemann-Liouville fractional integral operator we have the following results.

### Remark 3.4

In Theorem 3.2 for different values of the parameter, we have

(i) if we put $\omega=0$, then we get [18], Theorem 6;
(ii) for $\omega=0$, $\nu=\frac{\mu}{k}$ and $g(s)=1$, then we get [17], Corollary 2.3;
(iii) for $\omega=0$ and $\nu=1$, we get [18], Corollary 3.

Next we give the following result.

### Theorem 3.5

*Let* $f:I\rightarrow\mathbb{R}$ *be a differentiable function on* *I*, $a,b\in I$ *with* $a< b$ *and also let* $g:[a,b]\rightarrow\mathbb{R}$ *be a continuous function on* $[a,b]$. *If* $\vert f'\vert^{q}$, *where* $q>0$, *is a convex function on* $[a,b]$, *then the following inequality holds for* $k< l+\mu$:

$$\begin{aligned} &\biggl\vert \biggl( \int_{a}^{b}g(s)E_{\mu,\nu,l}^{\gamma,\delta,k} \bigl( \omega s^{\mu}\bigr)\,ds \biggr) ^{\nu}\bigl[f(a)+f(b) \bigr]-\nu \int_{a}^{b} \biggl( \int_{a}^{t}g(s)E_{\mu,\nu,l}^{\gamma,\delta,k} \bigl(\omega s^{\mu}\bigr)\,ds \biggr) ^{\nu-1} \\ &\qquad {}\times g(t)E_{\mu,\nu,l}^{\gamma,\delta,k}\bigl(\omega t^{\mu} \bigr)f(t)\,dt \\ &\qquad {} -\nu \int_{a}^{b} \biggl( \int_{t}^{b}g(s)E_{\mu,\nu,l}^{\gamma, \delta,k}\bigl( \omega s^{\mu}\bigr)\,ds \biggr) ^{\nu-1}g(t)E_{\mu,\nu,l}^{ \gamma,\delta,k} \bigl(\omega t^{\mu}\bigr)f(t)\,dt\biggr\vert \\ &\quad \leq\frac{2(b-a)^{\nu+1}\Vert g\Vert _{\infty}^{\nu}S^{\nu }}{(\nu p+1)^{ \frac{1}{q}}} \biggl[ \frac{\vert f'(a)\vert^{q}+\vert f'(b)\vert ^{q}}{2} \biggr] ^{ \frac{1}{q}}, \end{aligned}$$

*where* $\Vert g \Vert _{\infty}= \sup_{t\in[a,b]}\vert g(t)\vert$ *and* $\frac{1}{p}+\frac{1}{q}=1$.

### Proof

By using Lemma 3.1, we have

10 $$\begin{aligned} &\biggl\vert \biggl( \int_{a}^{b}g(s)E_{\mu,\nu,l}^{\gamma,\delta,k} \bigl( \omega s^{\mu}\bigr)\,ds \biggr) ^{\nu}\bigl[f(a)+f(b) \bigr]-\nu \int_{a}^{b} \biggl( \int_{a}^{t}g(s)E_{\mu,\nu,l}^{\gamma,\delta,k} \bigl(\omega s^{\mu}\bigr)\,ds \biggr) ^{\nu-1} \\ & \qquad {}\times g(t)E_{\mu,\nu,l}^{\gamma,\delta,k}\bigl(\omega t^{\mu} \bigr)f(t)\,dt \\ &\qquad {} -\nu \int_{a}^{b} \biggl( \int_{t}^{b}g(s)E_{\mu,\nu,l}^{\gamma, \delta,k}\bigl( \omega s^{\mu}\bigr)\,ds \biggr) ^{\nu-1}g(t)E_{\mu,\nu,l}^{ \gamma,\delta,k} \bigl(\omega t^{\mu}\bigr)f(t)\,dt\biggr\vert \\ &\quad \leq \int_{a}^{b}\biggl\vert \int_{a}^{t}g(s)E_{\mu,\nu,l}^{\gamma, \delta,k} \bigl(\omega s^{\mu}\bigr)\,ds \biggr\vert ^{\nu}\bigl\vert f'(t)\bigr\vert \,dt+ \int_{a}^{b} \biggl\vert \int_{t}^{b}g(s)E_{\mu,\nu,l}^{\gamma,\delta,k}\bigl( \omega s ^{\mu}\bigr)\,ds \biggr\vert ^{\nu}\bigl\vert f'(t)\bigr\vert \,dt. \end{aligned}$$

Using the Hölder inequality, we have

11 $$\begin{aligned} &\biggl\vert \biggl( \int_{a}^{b}g(s)E_{\mu,\nu,l}^{\gamma,\delta,k} \bigl( \omega s^{\mu}\bigr)\,ds \biggr) ^{\nu}\bigl[f(a)+f(b) \bigr]-\nu \int_{a}^{b} \biggl( \int_{a}^{t}g(s)E_{\mu,\nu,l}^{\gamma,\delta,k} \bigl(\omega s^{\mu}\bigr)\,ds \biggr) ^{\nu-1} \\ & \qquad {}\times g(t)E_{\mu,\nu,l}^{\gamma,\delta,k}\bigl(\omega t^{\mu} \bigr)f(t)\,dt \\ &\qquad {} -\nu \int_{a}^{b} \biggl( \int_{t}^{b}g(s)E_{\mu,\nu,l}^{\gamma, \delta,k}\bigl( \omega s^{\mu}\bigr)\,ds \biggr) ^{\nu-1}g(t)E_{\mu,\nu,l}^{ \gamma,\delta,k} \bigl(\omega t^{\mu}\bigr)f(t)\,dt\biggr\vert \\ &\quad \leq\biggl( \int_{a}^{b}\biggl\vert \int_{a}^{t}g(s)E_{\mu,\nu,l}^{ \gamma,\delta,k} \bigl(\omega s^{\mu}\bigr)\,ds\biggr\vert ^{\nu p}\,dt \biggr) ^{ \frac{1}{p}} \biggl( \int_{a}^{b}\bigl\vert f'(t)\bigr\vert ^{q}\,dt \biggr) ^{\frac{1}{q}} \\ &\qquad {}+ \biggl( \int_{a}^{b}\biggl\vert \int_{t}^{b}g(s)E_{\mu,\nu,l}^{\gamma,\delta,k}\bigl( \omega s^{\mu}\bigr)\,ds\biggr\vert ^{\nu p}\,dt \biggr) ^{\frac{1}{p}} \biggl( \int_{a}^{b}\bigl\vert f'(t)\bigr\vert ^{q}\,dt \biggr) ^{\frac{1}{q}}. \end{aligned}$$

Using $\Vert g \Vert _{\infty}= \sup_{t\in[a,b]}\vert g(t)\vert$ and absolute convergence of the generalized Mittag-Leffler function, we have

12 $$\begin{aligned} &\biggl\vert \biggl( \int_{a}^{b}g(s)E_{\mu,\nu,l}^{\gamma,\delta,k} \bigl( \omega s^{\mu}\bigr)\,ds \biggr) ^{\nu}\bigl[f(a)+f(b) \bigr]-\nu \int_{a}^{b} \biggl( \int_{a}^{t}g(s)E_{\mu,\nu,l}^{\gamma,\delta,k} \bigl(\omega s^{\mu}\bigr)\,ds \biggr) ^{\nu-1} \\ & \qquad {}\times g(t)E_{\mu,\nu,l}^{\gamma,\delta,k}\bigl(\omega t^{\mu} \bigr)f(t)\,dt \\ &\qquad {} -\nu \int_{a}^{b} \biggl( \int_{t}^{b}g(s)E_{\mu,\nu,l}^{\gamma, \delta,k}\bigl( \omega s^{\mu}\bigr)\,ds \biggr) ^{\nu-1}g(t)E_{\mu,\nu,l}^{ \gamma,\delta,k} \bigl(\omega t^{\mu}\bigr)f(t)\,dt\biggr\vert \\ &\quad \leq \Vert g\Vert _{\infty}^{\nu}S^{\nu} \biggl[ \biggl( \int_{a}^{b}\vert t-a\vert^{ \nu p}\,dt \biggr) ^{\frac{1}{p}}+ \biggl( \int_{a}^{b}\vert b-t\vert^{\nu p}\,dt \biggr) ^{\frac{1}{p}} \biggr] \biggl( \int_{a}^{b}\bigl\vert f'(t)\bigr\vert ^{q} \biggr) ^{ \frac{1}{q}}. \end{aligned}$$

Since $\vert f'(t)\vert^{q}$ is convex function, we have

13 $$ \bigl\vert f'(t)\bigr\vert ^{q}\leq \frac{b-t}{b-a}\bigl\vert f'(a)\bigr\vert ^{q}+ \frac{t-a}{b-a}\bigl\vert f'(b)\bigr\vert ^{q}. $$

Using (13) in (12), we have

14 $$\begin{aligned} &\biggl\vert \biggl( \int_{a}^{b}g(s)E_{\mu,\nu,l}^{\gamma,\delta,k} \bigl( \omega s^{\mu}\bigr)\,ds \biggr) ^{\nu}\bigl[f(a)+f(b) \bigr]-\nu \int_{a}^{b} \biggl( \int_{a}^{t}g(s)E_{\mu,\nu,l}^{\gamma,\delta,k} \bigl(\omega s^{\mu}\bigr)\,ds \biggr) ^{\nu-1} \\ & \qquad {}\times g(t)E_{\mu,\nu,l}^{\gamma,\delta,k}\bigl(\omega t^{\mu} \bigr)f(t)\,dt -\nu \int_{a}^{b} \biggl( \int_{t}^{b}g(s)E_{\mu,\nu,l}^{\gamma, \delta,k}\bigl( \omega s^{\mu}\bigr)\,ds \biggr) ^{\nu-1}g(t)E_{\mu,\nu,l}^{ \gamma,\delta,k} \bigl(\omega t^{\mu}\bigr)f(t)\,dt\biggr\vert \\ &\quad \leq \Vert g\Vert _{\infty}^{\nu}S^{\nu} \biggl[ \biggl( \int_{a}^{b}\vert t-a\vert^{ \nu p}\,dt \biggr) ^{\frac{1}{p}}+ \biggl( \int_{a}^{b}\vert b-t\vert^{\nu p}\,dt \biggr) ^{\frac{1}{p}} \biggr] \\ &\qquad {}\times\biggl( \int_{a}^{b}\frac{b-t}{b-a}\bigl\vert f'(a)\bigr\vert ^{q}+\frac{t-a}{b-a}\bigl\vert f'(b)\bigr\vert ^{q} \biggr) ^{\frac{1}{q}}. \end{aligned}$$

After simplification, we get the required result. □

### Remark 3.6

By giving particular values to the parameters in the generalized Mittag-Leffler function, several fractional integral inequalities can be obtained for corresponding fractional integrals. For example for the Riemann-Liouville fractional integral operator we have the following result.

### Corollary 3.7

*In Theorem* 3.5, *if we take* $\omega=0$ *and* $g(s)=1$, *then we have the following inequality for the Riemann*-*Liouville fractional integral operator*:

$$\biggl\vert \frac{f(a)+f(b)}{2}-\frac{\Gamma(\nu+1)}{2(b-a)^{\nu }}\bigl[I_{a ^{+}}^{\nu}f(b)+I_{b_{-}}^{\nu}f(a) \bigr]\biggr\vert \leq\frac{b-a}{(\nu p+1)^{ \frac{1}{p}}} \biggl[ \frac{\vert f'(a)\vert^{q}+\vert f'(b)\vert ^{q}}{2} \biggr] ^{ \frac{1}{q}}. $$

### Remark 3.8

For particular values of the parameters, Theorem 3.5 gives the following results.

(i) If we put $\omega=0$, then we get [18], Theorem 7.
(ii) If we put $\omega=0$, $\nu=1$, then we get [18], Corollary 4.
(iii) If we take $\omega=0$ along with $\nu=\frac{\mu}{k}$, then we get [17], Theorem 2.5.

The next result is the Hadamard type inequality for a generalized fractional integral operator.

### Theorem 3.9

*Let* $f:[a,b]\rightarrow\mathbb{R}$ *be a positive and convex function with* $0 \leq a< b$. *If* $f \in L[a,b]$, *then the following inequalities for the generalized fractional integral hold*:

$$\begin{aligned} f \biggl( \frac{a+b}{2} \biggr) \bigl( \epsilon_{\mu,\nu ,l,\omega',(\frac{a+b}{2})^{+}}^{\gamma,\delta,k}1 \bigr) (b) & \leq\bigl[ \bigl( \epsilon_{\mu,\nu,l,\omega ',(\frac{a+b}{2})^{+}}^{\gamma,\delta,k}f \bigr) (b) + \bigl( \epsilon_{\mu,\nu,l,\omega',(\frac{a+b}{2})^{-}} ^{\gamma,\delta,k}f \bigr) (a) \bigr] \\ &\leq\frac{f(a)+f(b)}{2} \bigl( \epsilon_{\mu,\nu,l,\omega ',(\frac{a+b}{2})^{-}}^{\gamma,\delta,k}1 \bigr) (a), \end{aligned}$$

*where* $\omega'=\frac{2^{\mu}\omega}{(b-a)^{\mu}}$.

### Proof

Since *f* is a convex function, we have

15 $$ f \biggl( \frac{x+y}{2} \biggr) \leq\frac{f(x)+f(y)}{2} $$

for $x,y\in[a,b]$.

Substituting $x=\frac{2-t}{2}a+\frac{t}{2}b$, $y=\frac{t}{2}a+ \frac{2-t}{2}b$ for $t\in[0,1]$, inequality (15) becomes

16 $$ 2f \biggl( \frac{a+b}{2} \biggr) \leq f \biggl( \frac{2-t}{2}a+\frac{t}{2}b \biggr) +f \biggl( \frac{t}{2}a+ \frac{2-t}{2}b \biggr). $$

Multiplying both sides of (16) by $t^{\nu-1}E_{\mu,\nu,l} ^{\gamma,\delta,k}(\omega t^{\mu})$ and integrating over $[0,1]$, we have

17 $$\begin{aligned} 2 &f \biggl( \frac{a+b}{2} \biggr) \int_{0}^{1}t^{\nu-1}E_{\mu,\nu,l} ^{\gamma,\delta,k}\bigl(\omega t^{\mu}\bigr)\,dt \\ &\quad \leq \int_{0}^{1}t^{\nu-1}E_{\mu,\nu,l}^{\gamma,\delta,k} \bigl( \omega t^{\mu}\bigr)f \biggl( \frac{2-t}{2}a+ \frac{t}{2}b \biggr)\,dt \\ &\qquad {} + \int_{0} ^{1}t^{\nu-1}E_{\mu,\nu,l}^{\gamma,\delta,k} \bigl(\omega t^{\mu}\bigr)f \biggl( \frac{t}{2}a+ \frac{2-t}{2}b \biggr)\,dt. \end{aligned}$$

Setting $u=\frac{2-t}{2}a+\frac{t}{2}b$ and $v=\frac{t}{2}a+ \frac{2-t}{2}b$ in (17), we have

$$\begin{aligned} &2 f \biggl( \frac{a+b}{2} \biggr) \int_{\frac{a+b}{2}}^{b}(b-v)^{\nu-1}E _{\mu,\nu,l}^{\gamma,\delta,k}\bigl(\omega' (b-v)^{\mu} \bigr)\,dv \\ &\quad \leq \int_{\frac{a+b}{2}}^{b}(b-v)^{\nu-1}E_{\mu,\nu,l}^{\gamma,\delta,k} \bigl(\omega' (b-v)^{\mu}\bigr)f(v)\,dv \\ &\qquad {} + \int_{a}^{\frac{a+b}{2}}(u-a)^{ \nu-1}E_{\mu,\nu,l}^{\gamma,\delta,k} \bigl(\omega' (u-a)^{\mu}\bigr)f(u)\,du, \end{aligned}$$

where $\omega'=\frac{2^{\mu}\omega}{(b-a)^{\mu}}$.

This implies

18 $$ f \biggl( \frac{a+b}{2} \biggr) \bigl( \epsilon_{\mu ,\nu,l,\omega',(\frac{a+b}{2})^{+}}^{\gamma,\delta,k}1 \bigr) (b)\leq\bigl[ \bigl( \epsilon_{\mu,\nu,l,\omega',(\frac {a+b}{2})^{+}}^{\gamma,\delta,k}f \bigr) (b) + \bigl( \epsilon_{\mu,\nu,l,\omega',(\frac{a+b}{2})^{-}} ^{\gamma ,\delta,k}f \bigr) (a) \bigr] . $$

On the other hand, convexity of *f* gives

19 $$\begin{aligned} f \biggl( \frac{2-t}{2}a+\frac{t}{2}b \biggr) +f \biggl( \frac{t}{2}a+ \frac{2-t}{2}b \biggr) &\leq \frac{2-t}{2}f(a)+\frac{t}{2}f(b)+ \frac{t}{2}f(a)+ \frac{2-t}{2}f(b) \\ &= f(a)+f(b). \end{aligned}$$

Multiplying both sides of (19) by $t^{\nu-1}E_{\mu,\nu,l} ^{\gamma,\delta,k}(\omega t^{\mu})$ and integrating over $[0,1]$, we have

20 $$\begin{aligned}& \int_{0}^{1}t^{\nu-1}E_{\mu,\nu,l}^{\gamma,\delta,k} \bigl(\omega t ^{\mu}\bigr)f \biggl( \frac{2-t}{2}a+ \frac{t}{2}b \biggr)\,dt+ \int_{0}^{1}t^{ \nu-1}E_{\mu,\nu,l}^{\gamma,\delta,k} \bigl(\omega t^{\mu}\bigr)f \biggl( \frac{t}{2}a+ \frac{2-t}{2}b \biggr) \\& \quad \leq\bigl[f(a)+f(b)\bigr] \int_{0}^{1}t^{\nu-1}E_{\mu,\nu,l}^{\gamma, \delta,k} \bigl(\omega t^{\mu}\bigr)\,dt. \end{aligned}$$

Setting $u=\frac{2-t}{2}a+\frac{t}{2}b$ and $v=\frac{t}{2}a+ \frac{2-t}{2}b$ in (20), we have

21 $$\begin{aligned}& \int_{a}^{\frac{a+b}{2}}(u-a)^{\nu-1}E_{\mu,\nu,l}^{\gamma,\delta,k} \bigl(\omega' (u-a)^{\mu}\bigr)f(u)\,du + \int_{\frac{a+b}{2}}^{b}(b-v)^{ \nu-1}E_{\mu,\nu,l}^{\gamma,\delta,k} \bigl(\omega' (b-v)^{\mu}\bigr)f(v)\,dv \\& \quad \leq \int_{a}^{\frac{a+b}{2}}(u-a)^{\nu-1}E_{\mu,\nu,l}^{\gamma, \delta,k} \bigl(\omega' (u-a)^{\mu}\bigr)\,du. \end{aligned}$$

This implies

22 $$ \bigl[ \bigl( \epsilon_{\mu,\nu,l,\omega',(\frac {a+b}{2})^{+}}^{ \gamma,\delta,k}f \bigr) (b) + \bigl( \epsilon_{\mu,\nu,l,\omega',(\frac{a+b}{2})^{-}}^{\gamma ,\delta,k}f \bigr) (a) \bigr] \leq\frac{f(a)+f(b)}{2} \bigl( \epsilon_{\mu,\nu,l,\omega ',(\frac{a+b}{2})^{-}}^{\gamma,\delta,k}1 \bigr) (a). $$

Combining (18) and (22) we get the result. □

### Corollary 3.10

*In Theorem* 3.9 *if we take* $\omega=0$, *then we get the following inequality for the Riemann*-*Liouville fractional integral operator*:

$$f \biggl( \frac{a+b}{2} \biggr) \leq\frac{2^{\nu}\Gamma(\nu+1)}{(b-a)^{ \nu}}\bigl[I_{(\frac{a+b}{2})^{+}}^{\nu}f(b)+ I_{(\frac{a+b}{2})^{-}} ^{\nu}f(a)\bigr] \leq\frac{f(a)+f(b)}{2}. $$

### Remark 3.11

On giving particular values to the parameters in Theorem 3.9, we have the following results.

(i) If we put $\omega=0$ and $\nu=1$, we get the classical Hadamard inequality.
(ii) If we put $\omega=0$, then we get Theorem 2.1.

## Conclusion

In Section 3, we give the generalizations of the Hermite-Hadamard type inequalities via generalized fractional integrals. Also we prove a version of the Hadamard inequality for convex functions via a generalized fractional integral operator. Being generalizations, the results of [16–19] have been obtained. The idea is extendable for *m*-convex, *p*-convex and other related classes of functions.
